# The New Path Forward for Prior Authorizations: Navigating Differences in the Accepted Standards of Care, Society Guidelines, and FDA-approved Indications

**DOI:** 10.1093/ofid/ofaf765

**Published:** 2025-12-17

**Authors:** Angel N Desai, Adriana Rauseo, Gerald N Rogan, Andrej Spec, George R Thompson

**Affiliations:** Division of Infectious Diseases, Department of Internal Medicine, UC-Davis Health, Sacramento, California, USA; Division of Infectious Diseases, Department of Internal Medicine, Washington University, St. Louis, Missouri, USA; Rogan Consulting, Sacramento, California, USA; Division of Infectious Diseases, Department of Internal Medicine, Washington University, St. Louis, Missouri, USA; Division of Infectious Diseases, Department of Internal Medicine, UC-Davis Health, Sacramento, California, USA; Department of Medical Microbiology and Immunology, UC-Davis, Davis, California, USA

**Keywords:** prior authorization, insurance approval, indications, appeals process, off-label

In this manuscript, we review and focus on how to improve the current prior authorization (PA) process for self-administered prescription drugs (outpatient in-home prescriptions). The current process that serves as checks and balances between the patient and their health insurance provider has long been scrutinized and carries considerable delays and administrative costs for the patient, provider, and healthcare payer. The PA process verifies whether the drug is a benefit of the patient's health insurance (healthcare) plan [1]. If a covered benefit, the PA process verifies the use is medically necessary, which may “second guess” the opinion of the prescribing physician.

Both subscribers and providers are frequently displeased when a prescription is denied and frustrated with the time demands required by the PA process. However, when a less expensive drug should be used as the first-line treatment, there is often insufficient evidence to support the use of the requested, more expensive drug as being more effective or appropriate for the subscriber's condition. In some cases, the physician's recommendation may also differ from the accepted standard of care. The safety and efficacy of the requested medication also must be reviewed and assurance the requested agent is appropriate to minimize impacts on available resources. The failure to ensure safety, efficacy, and appropriate usage should result in denial of a prescription drug and alternative treatment options should be pursued or provided.

Problematic in this process is the lack of conformity for some conditions between the treating physician's preferred treatment agent, the approved indications used by medical insurance plans (payers), and the ability to view these criteria by physicians and/or patients. PA approval is generally straightforward when U.S. Food and Drug Administration (FDA) indications for a condition exist (also known as “on-label” indication). However, FDA indications are not sought by pharmaceutical companies for all-possible diseases/conditions that may benefit from the use of a particular agent, thereby limiting the lines of use listed in package inserts.

However, physicians and healthcare providers may still prescribe an FDA-approved drug for an indication not included in its label (“off-label” prescribing). Typically, when a drug is initially developed for its labeled use, treatment trials progress through the path of least resistance: the largest number of patients that are likely to receive the drug following approval, and trials that will be completed rapidly allowing the drug to promptly enter the market. It is cost-prohibitive for approval to be sought for rare diseases or to study every conceivable patient population that may benefit from this medication. As a result, a drug developed for more common diseases that is used off-label to treat rare conditions requires a PA to obtain insurance payment, and in fact 25% of all U.S. prescriptions are used off-label [2], with this number even higher (19%–43%) for anti-infective agents [3].

During the PA process, a licensed physician typically guides the review process. A Pharmacy and Therapeutics committee determines inclusion versus exclusion based on clinical appropriateness, creation of internal disease treatment guidelines, and recommendations of utilization management to ensure safe use. Pharmacy and Therapeutics committee members may rely on medical information not included in the drug's label, such as published per reviewed research, clinical practice guidelines; formularies developed by other plans; independent review companies that publish drug compendia, such as Micromedex, American Hospital Formulary Service Drug Information, United States Pharmacopoeia–Drug Information, DRUGDEX Information System, NCCN guidelines; and other authoritative sources and formularies subsequently updated when additional information becomes available to payers. A financial team also may be involved to determine tiers (preferred, nonpreferred, etc), placement of preferred status versus what is deemed a clinically equivalent drug based off of negotiated manufacture rebates, and formulary exclusions of multiple drugs that share similarity (limiting to one). It is essential that physicians/prescribers are aware they may also inadvertently delay or hinder the PA process by submitting incomplete, or inaccurate information or while awaiting final confirmatory laboratory or radiographic information.

There may be tension during this review process because of differences in the relevant accepted standard of care, society guidelines, and FDA indications. Standard of care may rapidly evolve, whereas society guidelines often take years to be updated or are never updated, and FDA approval may never be pursued for certain indications.

An example of this process is prescriptions for subscribers with systemic fungal infections—a group frequently subject to the PA process given the potential need for off-label prescribing. The relative infrequency of invasive fungal infections apart from candidiasis, aspergillosis, and cryptococcosis has resulted in few randomized clinical trials, with FDA approval often not sought for less common indications despite high associated mortality. Without published evidence, medical necessity vests with expert opinions. Society guidelines exist for some of these diseases, including the endemic mycoses, rare molds, and yeasts, yet the PA approval process often fails to approve needed medications during the care of these patients. It is therefore infrequent that payers authorize off-label medical necessity; however, alternative agents are similarly not approved, leaving patients with no appropriate treatment option. The patient must then self-pay or appeal to a third party.

Further complicating this process, treatment guidelines may take years for specialty societies to develop. Older recommendations may not include recent scientific evidence or expert clinical experience. For example, coccidioidomycosis is a regional fungal infection caused by inhalation of spores within the endemic region. For treatment, the FDA has approved only amphotericin B deoxycholate and ketoconazole, with the latter being an outdated treatment option no longer recommended for systemic therapy for any infection [4], whereas the current standard of care to treat coccidioidomycosis includes the off-label use of triazole antifungal drugs (eg, fluconazole, itraconazole) and other agents frequently needed in those with no response or with toxicity to these agents (voriconazole, posaconazole, isavuconazonium) [5, 6]. Nonetheless, these drugs, particularly the latter prescribed for refractory disease or intolerance to alternatives, are often denied by the PA process because their off-label use is not supported by guidance documents for medical necessity. Likewise, off-label uses of drugs may be denied when prescribed to treat diseases with no FDA indication, yet with clinically accepted treatments that improve outcomes *Lomentospora, Scopulariopsis,* and other rare fungal diseases). Similar problems exist in the treatment of nontuberculous mycobacterial infections (eg, use of bedaquiline, tedizolid, omadacycline) and specific *Clostridium difficile* treatment options.

During the PA process for conditions such as these, insurance companies review the claims for drugs and decide whether their use is medically necessary or “experimental and investigational.” Typically, third-party administration contracts (an external company that handles the administrative functions [claims adjudication, network access, etc] for a self-funded health plan or employee benefit program) and individual policies specify no payment may be made for drugs and services that are considered “experimental and investigational.” Most problematic is the lack of alternative treatment options to be presented following denial, and the increasing lack of peer-to-peer availability in the PA process to discuss clinical cases.

Although many insurers do offer a dispute resolution process, those available are often cumbersome for the treating provider. A provider may assume an appeal will occur when a specific off-label use of a potentially life-saving drug has not been well-studied; however, for practical purposes, access to the drug may be denied for a patient without funds to self-pay while awaiting the results of an appeal, which may take months during the multiple appeal attempts and in some cases judicial proceedings—a process strongly biased against patient care.

## PROPOSED SOLUTIONS

### Inefficiency of Process

When the prescribing physician and the insurer disagree, a faster appeal process (<72 hours) coupled with a mechanism to reduce the need for appeals is critical. Peer-to-peer consultation is additionally essential during this process and must be made available to providers during the review process—we suggest as a mandatory component of appeals process to ensure patients are provided appropriate therapeutic agents.

### Availability of Updated Guidelines

Because of the absence of evidence granting FDA approval for some drug–disease combinations and the lack of continuous and real-time updates in authoritative guidelines for some infectious diseases, we propose relevant specialty societies work with plan medical directors to establish a reliable and accountable contemporary scientific evidence base that would be publicly available, used by PA reviewers, and sponsored by the Centers for Medicare and Medicaid Services as part of each guideline update process. State and Medicare plans may be targeted first. The United States Preventive Service Task Force as well as NCCN guidelines are just a few examples of how stakeholders can develop an up-to-date and agile evidence base. Crowdsourcing experts and the use of artificial intelligence can be used to add new articles and enable continuous updating of databases with recent evidence/publications. Fully published articles could additionally be added to the database with the permission of copyright holders.

### Guidance Documents

Once the scientific evidence is reviewed and expert opinions elucidated, a guidance document could be developed and published, with semiannual updates. With this approach, the PA process could be streamlined for the benefit of all concerned: properly denying off-label use that is not supported by the latest guidance, while removing barriers to pay for those off-label uses that are supported. A reasonable appeal mechanism could be established for experts who disagree with the guidance, both for a case-by-case review for specific patients, and for the guidance document itself. While reforming this process could provide access to more therapeutic options for patients with difficult to treat infections, PAs remain a significant challenge for a wide range of therapeutics across all medical specialties. Our experience is likely reflective of other areas of medicine that would similarly benefit from our proposed recommendations.
